# Chromatin Evolution and Molecular Drive in Speciation

**DOI:** 10.1155/2012/301894

**Published:** 2011-12-01

**Authors:** Kyoichi Sawamura

**Affiliations:** Graduate School of Life and Environmental Sciences, University of Tsukuba, 1-1-1 Tennodai, Tsukuba, Ibaraki 305-8572, Japan

## Abstract

Are there biological generalities that underlie hybrid sterility or inviability? Recently, around a dozen “speciation genes” have been identified mainly in *Drosophila*, and the biological functions of these genes are revealing molecular generalities. Major cases of hybrid sterility and inviability seem to result from chromatin evolution and molecular drive in speciation. Repetitive satellite DNAs within heterochromatin, especially at centromeres, evolve rapidly through molecular drive mechanisms (both meiotic and centromeric). Chromatin-binding proteins, therefore, must also evolve rapidly to maintain binding capability. As a result, chromatin binding proteins may not be able to interact with chromosomes from another species in a hybrid, causing hybrid sterility and inviability.

## 1. Introduction

Are there biological generalities that underlie hybrid sterility or inviability? In other words, do common mechanisms dictate that mules and leopons, for example, are sterile? The widely accepted Dobzhansky-Muller incompatibility (DMI) model of reproductive isolation [1, 2] does not provide an answer to this question. Instead, the DMI model only predicts that combinations of incompatible genes from different species lead to sterile or inviable hybrids. According to Mayr [3], reproductive isolation is an accidental byproduct of speciation. Recently, around a dozen “speciation genes” have been identified, and the biological functions of these genes are revealing molecular generalities that control hybrid sterility and inviability [4–8] (but see [9]). They are chromatin evolution and molecular drive in speciation.

Dover [10] argues, “In the case of many families of genes and noncoding sequences…, fixation of mutations within a population may proceed as a consequence of molecular mechanisms of turnover within the genome [i.e., molecular drive]. …There are circumstances in which the unusual concerted pattern of fixation permits the establishment of biological novelty and species discontinuities [i.e., reproductive isolation]…” Genes encoding heterochromatin proteins may have evolved rapidly to counteract mutations within repetitive DNA sequences in heterochromatin, which accumulate by molecular drive. The molecular drive theory once dominated the field of speciation, supported by the discovery that selfish transposable elements cause hybrid dysgenesis [11–14]. However, this hypothesis has been discounted, as there is no direct evidence that transposons are involved in reproductive isolation [15, 16] (but see [17, 18]). Even the most contemporary textbook concerning speciation [19] does not cite the Dover's [10].

## 2. *Lhr* and *Hmr* of *Drosophila*

When *Drosophila melanogaster* females mate with *Drosophila simulans* males, only weak, sterile, female hybrids eclose, as male hybrids die during larval stages [20]. Watanabe [21] discovered a *D. simulans* mutation, *Lethal hybrid rescue* (*Lhr*), that prevents hybrid larval lethality and restores female hybrid vigor [22]. It was thought that the wild-type allele of *D. simulans Lhr* was incompatible with X-linked genes from *D. melanogaster*. It has since been demonstrated that *Lhr* encodes a heterochromatin protein, HP3, which contains a boundary element-associated factor 32/Su(var)3-7/Stonewall (BESS) domain [23–25]. The X-linked *Hybrid male rescue* (*Hmr*) of *D. melanogaster* [26] has an effect similar to *Lhr* when mutated, and it also restores female hybrid fertility in this context [27]. *Hmr* encodes a DNA-binding protein with two myb/SANT-like in Adf-1 (MADF) domains [28].

LHR and HMR may physically interact through their BESS and MADF domains and may colocalize to specific chromatin regions. LHR also interacts with the heterochromatin proteins HP1 and HP6, as demonstrated by yeast two-hybrid (Y2H) experiments, RNA interference (RNAi) knockdown, and Bayesian network analysis [23, 25, 29–31]. The ratio of the number of nonsynonymous substitutions per nonsynonymous site to the number of synonymous substitutions per synonymous site (*K_a_/K_s_*) [32] and McDonald-Kreitman (MK) test [33] indicate that *Hmr* and a subset of genes encoding heterochromatin proteins (including *Lhr* and *HP6*) have evolved under positive selection [23, 28, 31, 34]. The involvement of *Lhr* and *Hmr* in reproductive isolation is reminiscent of speciation mediated by molecular drive. A comprehensive analysis of LHR, but not HMR, binding sites in the genome has been performed [35].

## 3. *zhr* of *Drosophila*

Involvement of heterochromatic repetitive sequences in hybrid inviability is evident when crosses between *D. simulans* females and *D. melanogaster* males (reciprocal to the cross discussed above) are analyzed. Progeny from this cross are sterile, male hybrids, as most female hybrids die during embryogenesis [20, 36]. We discovered *zygotic hybrid rescue* (*zhr*), a *D. melanogaster* gene that prevents female embryonic lethality in this context [37]. Genetic analyses using chromosome deficiencies and duplications [38–40] indicate that female hybrids are rescued if the number of 359-bp repetitive sequences (1.688 satellite) on the *D. melanogaster* X chromosome is decreased. In addition, hybrids of both sexes are inviable when repetitive sequences are added. In embryos from *D. simulans* mothers, chromatin regions rich in the 1.688 satellite are not properly condensed [41], resulting in mitotic defects such as chromosome bridges and irregularly spaced nuclei [41, 42].

The 1.688 satellite was one of the earliest sequences cloned in *Drosophila* [43, 44] and represents more than 4% of the *D. melanogaster *genome [45–47]. Related sequences are present in *D. simulans*, but the homology is low [48–51]. Heterochromatin regions rich in the 1.688 satellite may represent binding sites for the putative HMR/LHR complex. However, because *zhr* only affects hybrid viability when *D. simulans* females are crossed to *D. melanogaster* males (not the reciprocal cross), the larval and embryonic hybrid-inviability phenotypes associated with these crosses were thought to be independent (see [37, 52] for additional evidence). However, the possibility remains that female hybrids from *D. melanogaster* mothers are viable because proteins necessary to cope with *D. melanogaster* heterochromatin on the X chromosome are supplied maternally. This explanation is consistent with the model proposed by [53, 54]. Identification of proteins that bind to the 1.688 heterochromatin satellite will be informative [55–58]. *maternal hybrid rescue* (*mhr*) of *D. simulans* [52] and *Simulans hybrid females rescue* (*Shfr*) [59] represent loci encoding strong 1.688-binding candidates.

Although the 1.688 satellite does not seem to encode any proteins, it is transcribed in ovaries and silenced by the RNAi machinery. This silencing is mediated by repeat-associated small interfering RNA, also called Piwi-associated RNA [60]. In hybrids, failure to silence the 1.688 satellite may lead to heterochromatin decondensation and lethality [54]. Finally, the *hybrid lethal on the X* (*hlx*) locus of *D. mauritiana* affects viability of *D. simulans* hybrids and has been mapped to heterochromatin [61]. It will be interesting to determine whether this locus also consists of repetitive sequences, similar to *zhr*.

## 4. *OdsH* of *Drosophila*

In reciprocal crosses between *D. mauritiana* and *D. simulans*, female hybrids are fertile but male hybrids are sterile [62]. Many genes have been identified that affect this male hybrid sterility (for a review see [63]). These loci are scattered throughout the two genomes, but an X-linked gene, *Odysseus* (*Ods*), plays a particularly important role. When the *D. mauritiana* allele of *Ods* is cointrogressed with a closely linked gene onto the *D. simulans* genetic background, males become sterile [64, 65]. This hybrid male sterility gene has been isolated as *Ods-site homeobox* (*OdsH*) [66]. *OdsH* is paralogous to *uncoordinated-4* (*unc-4*), which is expressed in postmitotic neurons and epidermal cells [67]. In *Drosophila*, *OdsH* is thought to have arisen through gene duplication and neofunctionalization, thereby assuming a novel role in spermatogenesis [66, 68, 69]. Ample evidence suggests that *OdsH*, especially its DNA-binding homeodomain, has evolved under positive selection [66, 69]. Four genes downregulated in sterile male hybrids are thought to lie downstream of *OdsH* [70]. And misexpressed genes are disproportionately more common on autosomes than on the X in the males with *OdsH* introgression [71]. Regulatory regions of these genes may contain binding sites for the *OdsH* transcription factor.

Alternatively, but not mutually exclusively, Bayes and Malik [72] suggested that the ODSH protein localizes to evolutionarily dynamic loci in heterochromatin and that ODSH abundance and localization during premeiotic phases of spermatogenesis are different between *D. simulans* and *D. mauritiana*. ODSH from *D. mauritiana* associates with the heterochromatic Y chromosome of *D. simulans,* leading to decondensation and male hybrid sterility [72]. These data reveal that rapid heterochromatin evolution affects the onset of male hybrid sterility [72], in addition to hybrid inviability [37, 41]. However, it remains unclear which DNA sequences ODSH binds with the highest affinity.

## 5. *Nup160* and *Nup96* of *Drosophila*

The discovery of strains that restore the fertility of *D. simulans/D. melanogaster* female hybrids [73] provided the tools to introgress *D. simulans* chromosomal segments onto the *D. melanogaster* genetic background [74]. Both male and female introgression homozygotes successfully made were sterile, and the genes responsible for the male and female sterility have been mapped [75–77]. Among them, *Nucleoporin 160* (*Nup160*) of *D. simulans* was identified as the gene underlying female sterility on the *D. melanogaster* genetic background [78]. Both *D. simulans Nup160* and *Nucleoporin 96* (*Nup96*), which also encodes a component protein of the nuclear pore complex (NPC), cause inviability in *D. melanogaster/D. simulans* male hybrids [78–80]. This is independent of the F_1_ hybrid inviability that can be rescued by *Lhr* mutation and is only revealed in introgression bearers or hemizygotes made from *D. melanogaster* deficiencies [81, 82].

Population genetics studies have indicated that positive selection is operating in seven nucleoporin genes, including *Nup160* and *Nup96* [79, 80, 83] and have revealed significant correlated evolution between them [84]. Several hypotheses have been proposed for why nucleoporins are evolving so rapidly in *Drosophila* [78–80, 83], but here I will focus on the hypothesis most highly related to the molecular drive theory. The NPC forms channels that allow transport of macromolecules between the nucleus and cytoplasm (for a recent review see [85]). In addition, NPC components also function in kinetochore/spindle formation and transcriptional regulation (i.e., dosage compensation) [86–91]. The evolution of scaffold nucleoporins (the NUP107-160 complex) may have accelerated to recognize repetitive sequences in centromeric heterochromatin. In this way, incompatible NPCs may result in hybrid sterility and inviability through improper kinetochore formation. Alternatively, small RNAs derived from repetitive DNA sequences may not be properly trafficked in cells with incompatible NPCs. This leads to chromatin decondensation and, ultimately, sterility or inviability. Such a model has been proposed in the meiotic drive system of *D. melanogaster* (see below). In this case, mislocalized and truncated Ran GTPase Activating Protein (RanGAP), which is encoded by *Segregation distortion* (*Sd*) [92], disrupts proper nuclear transport of small RNAs derived from *Responder* (*Rsp*) and ribonucleoprotein complexes that are required to suppress the *Rsp* satellites [54, 93].

## 6. *Prdm9* of Mice

Evidence for chromatin mechanisms in speciation is not restricted to *Drosophila*. In the cross between *Mus musculus musculus* and *M. m. domesticus*, female hybrids are fertile, but male hybrids are sterile (for a review see [101]; see also [102, 103]). Backcross analyses have indicated that three or more independently segregating loci are involved in this male hybrid sterility. One gene, *Hybrid sterility 1* (*Hst1*) of *M. m. domesticus*, is polymorphic: the *Hst1^s^* allele causes sterility, but *Hst1^f^* does not [104]. This situation is similar to the hybrid rescue mutations in *Drosophila*. The *Hst1* locus was mapped to the *PR domain zinc finger protein 9* (*Prdm9*) gene, where PR stands for PRDIBF1 and RIZ homology. *Prdm9* encodes a histone H3 lysine 4 (H3K4) trimethyltransferase [94], which is also known as the *Meisetz*, meiosis-induced factor containing a PR/SET domain and a zinc-finger motif [105]. Hybrid males sterilized by the *Prdm9* introgression exhibit frequent dissociation of the X and Y chromosomes during meiosis [94], similar to the sterile male hybrid from a cross between *M. m. musculus* and *M. spretus* [106–108]. A gene involved in *M. musculus*/*M. spretus* male hybrid sterility and a gene responsible for X-Y dissociation in *M. m. musculus*/*M. m. molossinus* hybrid males (the latter termed *Sex-chromosome association* (*Sxa*)) have been mapped to the pseudoautosomal region of the X chromosome [95, 96]. The heterochromatin content of this region is quantitatively different among species or subspecies [109, 110].

The DNA-binding domain of PRDM9 consists of multiple, tandem C2H2 zinc finger domains and is evolving rapidly under positive selection in diverse metazoans, including rodents and primates. Rapid evolution of this binding domain likely results from recurrent selection for binding specificity to satellite DNAs [111–113]. The interaction between PRDM9 and repetitive sequences also affects meiotic recombination [114–116]. Histone H3 modifications are typical epigenetic events that determine chromatin status (for reviews see [117, 118]). Genomic regions characterized by heterochromatin-mediated gene silencing are rich in histone H3K9 methylation and have few histone acetylations. In contrast, histones in transcriptionally active euchromatic regions are highly acetylated and methylated at H3K4. Interestingly, chromatin structures regulated by H3K9 methylation, Su(var)3-9, HP1, or the RNAi pathway are required to maintain the structural integrity of tandemly repeated, heterochromatic sequences, like the 1.688 satellite, in *D. melanogaster* [119].

## 7. Three Drives in Speciation

The meiotic drive model of male hybrid sterility assumes an arms race between meiotic drive genes and suppressor genes in which male hybrids exhibit segregation distortion or sterility if they inherit drive genes, but not their corresponding suppressors [120, 121]. At first, this model was not accepted because cryptic segregation distortion was not detected in interspecies crosses of *Drosophila* [122, 123]. In the cross between *D. mauritiana* and *D. simulans*, one gene involved in male hybrid sterility is not separable from the meiotic drive gene, *too much yin* (*tmy*), by recombination [97]. In addition, the gene *Overdrive* (*Ovd*) causes both male hybrid sterility and meiotic drive in aged males when *D. pseudoobscura pseudoobscura* is crossed with *D. p. bogotana* [98, 124]. Interestingly, *Ovd* encodes a protein that contains a MADF DNA-binding domain [98], similar to HMR of *D. melanogaster* [28].

In the context of speciation, meiotic drive can be the manifestation of molecular drive. The most common example of this phenomenon is centromere drive. The centromere drive model assumes that both DNA and protein components of centromeric chromatin are evolving rapidly and that incompatibilities between rapidly evolving centromeric components may be responsible for hybrid sterility [125]. In particular, the expansion of centromeric repetitive sequences provides more microtubule attachment sites, thereby creating a stronger centromere that tends to be included in the oocyte nucleus [125]. This represents an alternative force from molecular drive that is distinct from a variety of mutational processes that include replication slippage, unequal exchange, transposition, and excision [10, 126–128]. To suppress potential nondisjunction of chromosomes that carry expanded satellite DNAs, the gene *centromere identifier *(*cid*) has evolved rapidly in diverse organisms including *Drosophila* [129, 130]. *cid* encodes centromeric histone H3-like, a homologue of human Centromere protein A (CENP-A). Examples of centromeric repeats affecting meiotic drive include the *Rsp* locus of *D. melanogaster*, which is the target of *Sd* [131], and the Cent728 repeat, which is responsible for female meiotic drive in the Monkeyflower hybrid between *Mimulus guttatus* and *Mimulus nasutus* [99].

## 8. Applicability and Related Issues

Above I proposed a theory that hybrid sterility and inviability are generally the manifestation of chromatin evolution and molecular drive in the context of speciation, but I do not claim that this model explains every case. Among hybrid incompatibility genes discussed in recent review papers, only 10 of 18 (Table  1 of [5]), 8 of 14 (Table  1 of [6]), and 7 of 14 (Table  S1 of [9]) are consistent with this theory. In addition, as most hybrid incompatibility data are from *Drosophila*, a different trend may appear if reproductive isolation genes are identified from diverse taxa. A famous exception to this theory involves the *JYalpha* gene in *Drosophila*. *JYalpha* encodes a protein with sodium/potassium-exchanging ATPase activity and is located on chromosome 4 in *D. melanogaster* but on chromosome 3 in *D. simulans*. Therefore, males carrying homozygous introgression of *D. simulans* chromosome 4 on the *D. melanogaster* genetic background are sterile, as they do not inherit *JYalpha* from either species [100, 132, 133]. This is an example of male hybrid sterility caused by gene transposition between species, which is consistent with the gene duplication and nonfunctionalization model of speciation [134].

Haldane's rule is generally observed when hybrid sterility and inviability are encountered. This rule states that “when in the F_1_ offspring of two different animal races one sex is absent, rare, or sterile, that sex is the heterozygous [heterogametic (XY or ZW)] sex” [135]. This rule is empirical and seems to be a composite phenomenon [136–138], although the dominance theory is applicable in most cases [139]. Here I propose an additional explanation for Haldane's rule, based on chromatin evolution and molecular drive in speciation. In hybrid animals, chromatin-binding proteins supplied from one species may not be able to recognize the other species' Y or W chromosome, as these chromosomes are generally heterochromatic and have high levels of repetitive satellite DNAs. This results in meiotic or mitotic chromosome decondensation or nondisjunction and leads to hybrid sterility or inviability in the heterogametic sex.

There are several chromatin state systems that have not been discussed yet, which may be related to the present issue. First, inactivation of the X chromosome in primary spermatocytes is necessary for the normal progression of spermatogenesis in heterogametic (XY) males [140] (but see [141, 142]), a process termed meiotic sex chromosome inactivation (MSCI). In some cases, male hybrid sterility may result from ineffective MSCI, as DNA-binding proteins may not be able to recognize and inactivate X chromosomes from different species (e.g., [63, 108]). Second, genomic imprinting affects a subset of genes, resulting in monoallelic and parent-of-origin-specific expression. This process usually depends on DNA methylation or histone modification (e.g., [143–146]). Species-specific variations in epigenetic marks may disrupt imprinting and lead to hybrid inviability. This can explain classic observations of unilateral incompatibility in rodent and flowering plant species (e.g., [147–150]).

## 9. Conclusion

**Figure 1:**
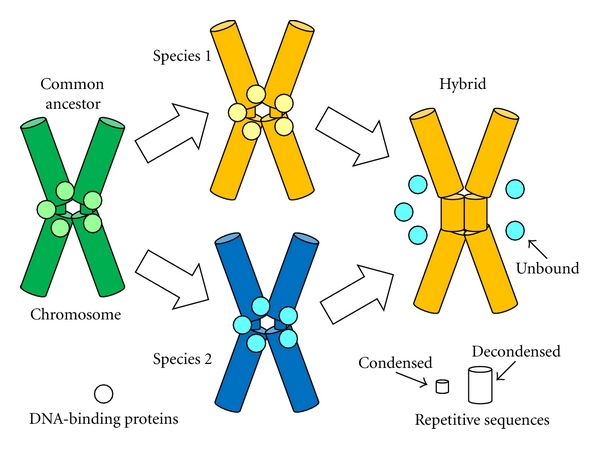
A hybrid sterility and inviability model based on chromatin evolution and molecular drive in speciation. Repetitive satellite DNAs evolve rapidly, thereby accelerating the evolution of chromatin-binding proteins (from the common ancestor to species 1 and species 2). Hybrids are sterile or inviable because the chromatin-binding proteins from species 2 cannot recognize the repetitive sequences of species 1.

As has been discussed in this paper, major cases of hybrid sterility and inviability seem to result from chromatin evolution and molecular drive in speciation (Table 1). Repetitive satellite DNAs within heterochromatin, especially at centromeres, evolve rapidly through molecular drive mechanisms (both meiotic and centromeric). Chromatin-binding proteins, therefore, must also evolve rapidly to maintain binding capability. As a result, chromatin-binding proteins may not be able to interact with chromosomes from another species in a hybrid, causing hybrid sterility and inviability (Figure 1).

## Figures and Tables

**Table 1:** Hybrid incompatibility genes mentioned in the current paper. Whether data concerning these genes are consistent or inconsistent with the current hypothesis is indicated.

Gene	Species	Phenotype^a^	Comment	Consistent	Reference
*Lhr* (HP3)	*Drosophila melanogaster/D. simulans*	F_1_-L	Interaction with heterochromatin proteins	Yes	[23, 24]
*Hmr*	*D. melanogaster/D. simulans*	F_1_-L, FS	Chromatin-binding	Yes	[28]
*zhr* (1.688)	*D. melanogaster/D. simulans*	F_1_-L	Centromeric repetitive DNA	Yes	[37, 41]
*hlx*	*D. melanogaster/D. simulans*	BC-L	Centromeric repetitive DNA?	Yes	[61]
*OdsH*	*D. melanogaster/D. simulans *	F_1_, BC-MS	Heterochromatin-binding	Yes	[66, 72]
*Nup160*	*D. melanogaster/D. simulans*	BC-L, FS	Centromeric heterochromatin-binding?	Yes	[78, 80]
*Nup96*	*D. melanogaster/D. simulans*	BC-L	Centromeric heterochromatin-binding?	Yes	[79]
*Prdm9*	*Mus m. musculus/M. m. domesticus*	F_1_, BC-MS	Histone methylation	Yes	[94]
*Sxa*	*M. m. musculus/M. m. domesticus; M. musculus/M. spretus*	F_1_, BC-XY, MS	Heterochromatic repetitive DNA?	Yes	[95, 96]
*tmy*	*D. simulans/D. mauritiana*	BC-MS	Not separable from the gene causing meiotic drive	Yes	[97]
*Ovd*	*D. p. pseudoobscura/D. p. bogotana*	F_1_, BC-MS	Chromatin-binding; also causing meiotic drive	Yes	[98]
Cent728	*Mimulus guttatus/M. nasutus*	F_1_, BC-FMD	Centromeric repetitive DNA	Yes	[99]
*JYalpha*	*D. melanogaster/D. simulans*	BC-MS	Transposition	No	[100]

^a^F_1_: hybrid; BC: (equivalent to) backcross; L: lethal; FS: female sterile; MS: male sterile; XY: XY dissociation; FMD: female meiotic drive.
